# The Influence of Steroid Hormones on Tooth Wear in Children and in Adolescents

**DOI:** 10.3390/jcm11133603

**Published:** 2022-06-22

**Authors:** Jeanette Buchhardt, Wieland Kiess, Antje Körner, Ronald Biemann, Christian Hirsch, LIFE Child Study Team

**Affiliations:** 1 Department of Pediatric and Preventive Dentistry, University of Leipzig, Liebigstr. 12, 04103 Leipzig, Germany; christian.hirsch@medizin.uni-leipzig.de; 2 Department of Women and Child Health, Hospital for Children and Adolescents and Center for Pediatric Research (CPL), University of Leipzig, Liebigstr. 20a, 04103 Leipzig, Germany; wieland.kiess@medizin.uni-leipzig.de (W.K.); antje.koerner@medizin.uni-leipzig.de (A.K.); 3 LIFE Leipzig Research Center for Civilization Diseases, University of Leipzig, Philipp-Rosenthal-Str. 27, 04103 Leipzig, Germany; lifechild@medizin.uni-leipzig.de; 4 Institute of Laboratory Medicine, Clinical Chemistry and Molecular Diagnostic, University of Leipzig, Paul-List-Str. 13-15, 04103 Leipzig, Germany; ronald.biemann@medizin.uni-leipzig.de

**Keywords:** steroid hormones, testosterone, tooth wear, attrition, wear facets

## Abstract

(1) Background: From a young age, boys are more often affected by tooth wear than girls. This suggests an influence of the male sex hormone (testosterone) on the aetiology of tooth wear. The aim of the present study was to investigate the incidence of tooth wear in relation to steroid hormone levels in children. (2) Methods: 1022 test persons aged between 10 and 18 (491 male, 531 female) from the LIFE Child study underwent medical and dental examination. Tooth wear was measured through clinical inspection. Blood samples were taken to determine hormone levels (testosterone, SHBG). The level of free testosterone was calculated from the ratio of testosterone to SHBG. Using multivariable methods, the incidence of tooth wear was analyzed as a function of hormone levels, while controlling for confounders such as age, sex, social status, and orthodontic treatment. (3) Results: The incidence of tooth wear increased with age in both sexes. Boys showed significantly more often attrition facets than girls (17.5% vs. 13.2%, *p* < 0.001). Subjects with tooth wear showed significantly higher free testosterone levels than those without (males: *p* < 0.001, females: *p* < 0.05). After controlling for confounding variables, the risk of tooth wear increased by approximately 30.0% with each year of life (odds ratio [OR]boys = 1.29, 95% confidence interval [CI] = 1.04–1.56; [OR]girls = 1.32, 95% CI = 1.08–1.61). In addition, the risk of tooth wear increased by 6.0% per free testosterone scale score only in boys (OR = 1.06, 95% CI = 1.01–1.12). (4) Conclusions: Tooth wear is common in children and in adolescents, and it increases steadily with age in both sexes. The stronger increase and the higher prevalence among male adolescents can be explained by the additional effect of free testosterone.

## 1. Introduction

As caries experiences throughout young adults are considerably declining, the daily practicing dentist is becoming more focused on treating abnormal degrees of tooth wear [1]. Attrition represents one of the three types of tooth wear, and it describes unnatural tooth contact, e.g., caused by bruxism [2]. The generic term tooth wear stands for the loss of tooth surface that is not provided by caries [3]. Besides attrition, reasons for tooth wear are erosion (chemical dissolution) or abrasion (foreign objects such as needles, thread, and pens) [2]. Current evaluation systems such as the TWES (Tooth Wear Evaluation System) [2] use the criteria of Gandara and Truelove [4] to describe tooth wear qualitatively and quantitatively.

The loss of tooth structure represents a clinical sign of bruxism, next to fractures of teeth or restorations, hypersensitive or painful teeth, masticatory muscle hypertrophy, and others [5]. The loss of tooth structure is not in itself sufficient to make a definitive diagnosis of bruxism [6]. For a correct evaluation of bruxism, two major options are commonly used, however there is no universal consensus as yet—especially concerning specific tools and data collection [7]. First, there is a non-instrumental approach through questionnaires and clinical inspection and, second, an instrumental approach that is reached through electromyography or app-based assessments [8]. Besides detecting sleep bruxism, anamnestic questions about teeth grinding at night can also be indicative of other sleep problems, as a study by Segu et al. recently showed. According to this, there is significant evidence that parent-reported tooth grinding (sleep bruxism) correlates with several sleep disorders such as bedtime problems, night awakenings, snoring, nightmares, sleep talking, or difficulty waking up in the morning [9]. According to the evaluation system, Lobbezoo et al. 2018 proposes to graduate the diagnose of bruxism into three subtypes [8]. First, possible bruxism that is identified through clinical inspection; second, probable bruxism proven by clinical inspection with or without positive self-reports; and, third, definitive bruxism based on an instrumental approach with or without a positive self-report and/or clinical inspection [8].

Due to past studies perceiving that bruxism is not solely teeth grinding or clenching, an international team decided on a new universal definition in 2013. There, it was added that bruxism can, as well or exclusively, describe the bracing or the thrusting of the mandible [10]. Bruxism occurs either as rhythmic or non-rhythmic masticatory muscle activity during sleep (sleep bruxism) or as repetitive or sustained tooth contact and/or bracing or thrusting of the mandible during the day (awake bruxism) [10]. Data on the frequency of bruxism is diverse. Among children and adolescents, the incidence range is 3 to 49% regarding sleep bruxism [11] and 12.4 [12] to 34.5% [13] concerning daytime bruxism. Observations of sex differences among bruxing children are, again, varying. While some authors describe that boys are affected more frequently than girls [2,14,15,16,17,18], some see no differences between the sex distributions [19,20].

Bruxism’s importance, as with any other health condition, is determined by its impact on patients, i.e., its quality-of-life influence and how often this impact occurs and lasts. Indeed, bruxism is one of the most frequent chronic oral health conditions, lasting over substantial time periods and even spanning one’s lifetime [11,12,13,21]. Not only is bruxism a frequent phenomenon, it also affects all components of a patient’s oral health experience, i.e., all four oral health-related quality of life dimensions (OHRQoL) Oral Function, Orofacial Pain, Orofacial Appearance, and Psychosocial Impact [22,23]. The impact originates from three major mechanisms. First, bruxism, through its effect on tooth substance and tooth loss, affects all four OHRQoL dimensions. Second, the damaging influence on the longevity of oral restorations and tooth replacement is also four-dimensional. Third, functional, painful, and psychosocial impacts originate from bruxism’s detrimental effects on jaw muscles and jaw joints. Bruxism’s aetiology is multifactorial. Initially, morphological factors such as the patient’s occlusion were assumed to be major factors. Upon more research and understanding, emphasis switched from local factors, originating from the stomatognathic system, to general factors associated with the patient’s psychosocial situation. In conjunction, hormonal factors were found to be influential as well. For example, the hormone level of cortisol showed higher concentrations among bruxists [24,25], and catecholamine has also been linked to bruxism [26]. In addition, the central dopaminergic system seems to be associated with bruxism [27,28]. Hormone levels are intertwined with age and sex, another set of etiological factors for bruxism. However, the age influence has rarely been studied. While age was included in some studies of adult subjects and results are mixed here, only a very limited number, e.g., Castelo et al. [27] as well as Vanderas et al. [26], studied hormonal factors and the relationship with age in children—despite the fact that initial findings demonstrated evidence for an association. Bruxism can occur on its own but also together with comorbidities, e.g., behavioral problems, such as ADHS (attention deficit hyperactivity disorder) [15,29,30]; psychological factors (e.g., anxiety and stress) [31,32]; or obstructive sleep apnoea [33]. Further, drugs, smoking, alcohol use, genes, various diseases, media consumption, somniloquia, snoring, and factors reducing sleep quality, such as noise, light or less sleep time, are listed as risk factors for clinical bruxism [34].

Studying the hormone–bruxism relationship in children and in adolescents offers a unique opportunity because hormone levels change substantially over one’s lifetime. Unfortunately, as pointed out above, only a limited number of studies exist, and those that do exist typically suffer from methodological problems. On the one hand, the evaluation system is not yet fully accepted as knowledge about bruxism and its aetiology is still improving. In most cases, bruxism is measured by self-report or tooth wear [10]. On the other hand, the sample size is often small and important confounding as well as modifying factors for the hormone–bruxism relationship are not included [27]. For example, orthodontic treatment, which frequently occurs among youths, represents an important local factor for bruxism but has yet to be studied [35]. Based on bio-psychosocial disease understanding, it can be expected that local factors of the stomatognathic system interact with central, systemic factors. Consequently, these local factors need to be studied together with the central factors in a large sample of children and adolescents of both sexes, covering a wide age range.

The present study aimed to investigate the relationship between the blood levels of sex hormones and attrition facets in male and female children and in adolescents, while considering potential confounders (e.g., age, body mass index, socioeconomic status, and orthodontic treatment).

## 2. Materials and Methods

### 2.1. Study Subjects and Setting for Examination

**Figure 1:**
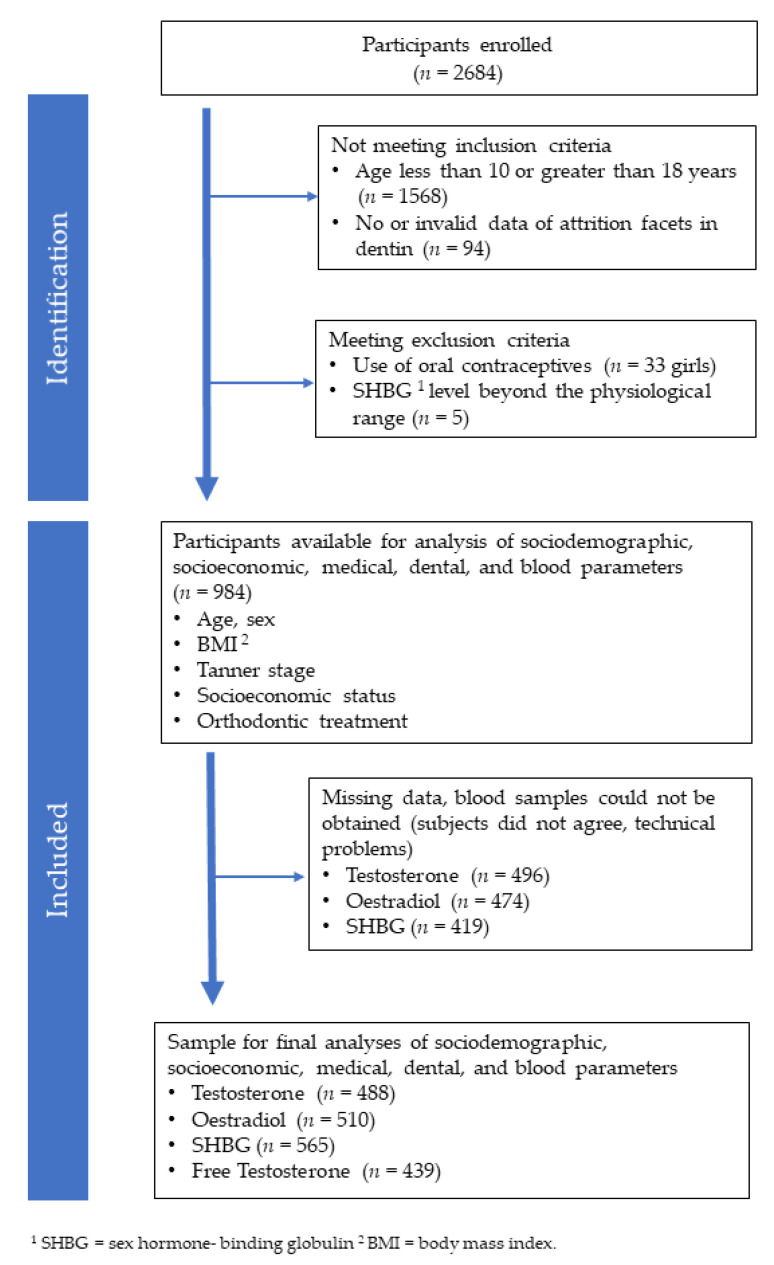
CONSORT flow diagram of study design and running.

The LIFE Child study (clinical trial number: NCT02550236) is a part of the “Research Center for Civilization Diseases”, and it is a large epidemiological as well as population-based cohort study of the University of Leipzig. The dental examination was approved by the Ethics Committee of the University of Leipzig (registration number: 354-10-13122010), and it took place between May 2012 and August 2015. This study includes 1022 participants between the ages of 10 and 18 years (491 male, 531 female), who were medically and dentally examined [Figure 1]. From the total sample, 33 girls using oral contraceptives as well as 5 test persons comprising an invalid (out of range) sex hormone-binding globulin (SHBG) level were excluded. This resulted in a net sample of 984 children and adolescents (491 male, 493 female) for the final analyses of sociodemographic, socioeconomic, medical, and dental variables. Blood samples for analyses of hormone levels were further collected from 488 (247 male, 241 female) test persons providing the testosterone level, 510 (258 male, 252 female) test persons providing the oestradiol level, 565 (279 male, 286 female) test persons providing the SHBG level, and 439 (215 male, 224 female) test persons providing the level of free testosterone. The free androgen index was calculated from the testosterone level in nmol/l multiplied by 100 and then divided by the immunoassayed SHBG level in nmol/l [36].

Before starting the medical and the dental examinations, parents and participants received information about the aim and the procedure, obtaining from them written approval. The test persons or guardians filled in a questionnaire with information about general health, medical history, socioeconomic status, and well-being. The survey took place in the study center from 2012 until 2015 by calibrated dentists according to standardized procedures. The oral health assessment was performed in a dental chair, with the use of a halogen light, a blunt probe, compressed air, and a mirror. The clinical data, such as orofacial pain, attrition, and orthodontic treatment, were collected with the help of a data processor. Wisdom teeth were not included in the assessment.

### 2.2. Determination of Outcome Variables

The outcome of interest was dental attrition (tooth wear). It was assessed for all anterior teeth (incisors and canines of maxilla and mandible). First, qualification of the tooth wear took place according to the clinical appearance described by Gandara and Truelove [2,4]: shiny, glossy, and flat facets; enamel and dentin wear at the same rate; matching wear on occluding surfaces, corresponding features at the antagonistic teeth; possible fracture of cusps or restorations; and impressions in cheek, tongue, and/or lip. Signs such as sensitivity and/or pain, absence of plaque, tartar, or staining served as indicators for present activity [2].

If attrition was determined, quantification of the tooth wear according to a modified version described by Egermark-Eriksson (1982) [37] followed. Tooth wear was diagnosed when dental attrition in enamel was larger than 1 mm^2^ and antagonistic teeth showed corresponding wear facets. Tooth wear was dissected for further analyses by means of two categories: the category “tooth wear” corresponded to grade 1 to 4 (wear in enamel and/or exposed dentin and/or loss of crown height) of the TWES (Tooth Wear Evaluation System) [2]. All subjects who had no tooth wear, as described by grad 0 of the TWES graduation, were assigned to the category “No tooth wear”.

In a former study [38], the inter-examiner reliability to assess tooth wear in a comparable study setting was excellent (intraclass correlation coefficient for test-retest-reliability = 0.92) according to guidelines [39].

### 2.3. Influential Variables

Venous blood serum was taken from the test persons to analyze hormone levels. The blood was processed by the LIFE pre-analytical laboratory of the LIFE Biobank and then immediately transferred to the Institute for Laboratory Medicine of the University Hospital Leipzig. A fully automated clinical chemistry analyzer (Cobas 8.000 Modular Analyzer) was used for the immunoassay analyses of testosterone, oestradiol, and sex hormone-binding globulin.

#### 2.3.1. BMI

The body mass index (BMI = weight [kg]/height [m]^2^) classified the nutritional condition of the subjects. The adjusted BMI was used to represent the deviation from the norm [40]. Negative values showed a deviation below the norm, whereas positive values were configured equal to or above the norm.

#### 2.3.2. SES

Socioeconomic status (SES) was assessed using the Winkler Index [41] that distinguishes between low (category 1), medium (category 2), or high (category 3) status.

#### 2.3.3. Pubertal Development

Pubertal development was evaluated using the 5-level Tanner scale [42] (secondary sex characteristics). The value of 1 represents the lowest pubertal development stage (no signs of secondary sex characteristics) and 5 the highest pubertal development stage (completed secondary sex characteristics).

#### 2.3.4. Orthodontic Treatment

Orthodontic treatment was determined and divided into the categories “Yes” (orthodontic appliance in past or present) or “No” (no appliance in past or present).

### 2.4. Statistical Analysis

The incidence of dental attrition (incisal tooth wear) was presented separately regarding the sexes of the test persons. Based on the absence or the presence of tooth wear, each group of female and male participants was then split in two categories. Firstly, the subjects of each sex were compared according to their category (tooth wear yes/no) regarding age, adjusted BMI, Tanner stage, socioeconomic status, orthodontic treatment, and hormone levels. Statistical tests were then performed: the *t*-test concerning continuous variables (age, BMI, Tanner stage, and hormone levels) and the chi-square test concerning categorical variables (high socioeconomic status, adjusted BMI, orthodontic treatment). Secondly, multivariable logistic regressions were performed to analyze the association between incisal tooth wear and the sex hormone level after controlling for the influence of confounding variables. All analyses were performed using IBM SPSS Statistics 24.0 software (IBM Corp, Armonk, NY, USA)

## 3. Results

### 3.1. Description of the Study Population

**Figure 2:**
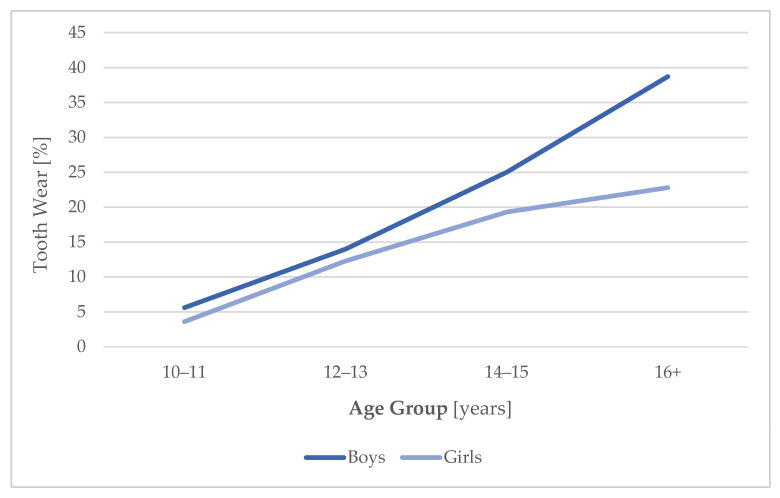
Age course of tooth wear prevalence in both sexes.

In this study, 491 boys and 493 girls, between the ages of 10 and 18, were included in the analyses. In total, 15.3% of all participants (*n* = 984) were affected by tooth wear (*n* = 151). Among the 10- to 13-year-old children (*n* = 620), a tenth (9.4%, *n* = 58) were affected by tooth wear, whereas it applied to one quarter (25.5%, *n* = 95) of the youth aged 14 and older (*n* = 364). A constantly higher percentage of boys with dental attrition than girls was observed. Among the male test persons (*n* = 491), 17.5% were affected by tooth wear (*n* = 86). Among the female test persons (*n* = 493), 13.2% were affected by tooth wear (*n* = 65). Additionally, the respective percentage increases at a stronger rate among the male participants [Figure 2]. Tooth wear applies to 10.3% (*n* = 33) of the 10- to 13-year-old and 31.0% (*n* = 53) of the 14- to 18-year- old boys. Concerning girls, the incidence of attrition facets shows a constant rise from 8.3% (*n*= 25) of the 10- to 13-year-olds to 20.7% (*n* = 40) of the 14-year-old and above group. A statistical significance of *p* < 0.001 is given for both sexes.

### 3.2. Comparison of Potentially Influencing Factors on Tooth Wear

Regardless of the sex, test persons with attrition facets showed significantly higher ages and higher Tanner stages [Table 1]. Further, the serum level of testosterone, oestradiol, SHBG, and free testosterone was significantly higher among male test persons with tooth wear than without (*p* < 0.001). Among female subjects with tooth wear, the serum level of testosterone and free testosterone was significantly higher than without (*p* < 0.05). The body mass index and the socioeconomic status score did not differ between children with or without attrition facets. Additionally, a deviation between the number of children with or without tooth wear under orthodontic treatment could not be seen.

### 3.3. Multivariable Analyses

Multivariable analyses reveal a significant increase in tooth wear with age in both sexes as well as an additional effect of testosterone in males [Table 2]. The odds ratio for tooth wear increases to 1.29 (95% confidence interval [CI]: 1.04–1.59) in males and 1.32 (95% CI: 1.08–1.61) in females per year. After controlling for the confounding variables, the risk of tooth wear was heightened by 6.0% of each scale value concerning the free testosterone measurement for boys (OR = 1.06; 95% CI: 1.01–1.12). Other variables such as BMI or orthodontic treatment did not show any effect. The status of pubertal development (Tanner stage) was deleted from the models because of the high correlation to age (r > 0.8).

## 4. Discussion

The present study aimed to answer the question of whether the incidence of tooth wear was attributed to the higher sex hormone levels of children and/or adolescents. In the multivariable analyses, the prevalence of tooth wear as a cumulative lifetime experience increased with age in both sexes. Amongst boys, an additional effect of free testosterone on tooth wear was observed, that is, the higher the hormone level, the more frequently attrition facets were present. Because dentists are not able to influence the hormonal status of their patients, the options for dental therapy based on this knowledge are limited. However, we now have a better understanding of the aetiopathogenesis as well as another diagnostic marker for bruxism. Furthermore, it is a possible explanation for the sex differences in bruxism among juveniles. These considerations contribute positively to the dental consultation with patients and the current scientific debate on bruxism.

Until now, there were no studies that considered the influence of sex hormone levels on wear facets amongst children and adolescents. There are several reasons for this. First, there are ethical difficulties in obtaining blood samples. The present study was embedded in a large project (LIFE Child study) in which, among other things, blood was obtained [43,44]. No blood samples were exclusively gained for the analyses. The procedure was approved by the Ethics Committee of the University of Leipzig (registration number: 354-10-13122010). Second, the blood collection itself posed challenges. Venous blood collection is a stressing process for which children and adolescents must be properly prepared [45]. In the present study, some test persons refused to have their blood drawn. Therefore, a sufficient volume of venous blood was obtained only from approximately one half of the subjects. Alternative examination procedures, e.g., the use of a finger stick, does not provide the amount of venous blood needed to determine an exact hormone level. Apart from this, differences in some parameters have been ascertained between venous and capillary blood. Blood provided by the center of the fingertip tended to show higher hormone levels than the blood drawn from the vein [46]. Due to its easier realizability, many studies observing the impact of hormones on bruxism among children and adolescents prefer to implement hormones that can be isolated from the saliva, for example cortisol [27,47,48,49], or from the urine, such as catecholamine [26]. Other than that, it is possible to use hair to measure hormone levels. However, the latter holds some disadvantages as, for example, hair shows cumulative hormone concentration across longer periods of time [50]. When weighing up the advantages and the disadvantages, hormone determination from venous blood currently seems to be the most sensible approach for studying the association between hormones and tooth wear.

Tooth wear is a cumulative lifetime experience. The observed attrition may have taken place in the past or may still be ongoing in the present.It has been associated with the hormone status of individuals. However, in an earlier publication we were able to show that sex hormone status stays constant for at least 2 years concerning the 10- to 18-year-old subjects [51]. So, we can assume that it is not a one-off hormone level. Therefore, it is reasonable to correlate both variables, tooth wear and hormone status, with each other as long-lasting phenomena. However, it was not investigated whether the subjects had already experienced bruxism in the primary dentition.

The universal comparability of the results is partly limited due to the following reasons. First, there is a wide range of grading scales for tooth wear. In the present study, a modified scale described by Egermark-Eriksson (1982) [37] is employed in order to quantify the severity of the tooth wear. It was used because of its adaptability and its ease of use, that is, the scale can be applied to any tooth, and it allows a largely error-free application. Further, studies applying the quantification score recommended by Wetselaar and Lobbezoo in 2016 [2] could improve universal comparability. Since the data were already collected before this recommendation, the corresponding classification of Wetselaar and Lobbezoo is additionally assigned in the present survey. Second, immunoassay tests show inaccuracies, especially measuring low hormone concentrations among females and prepubertal males as they occur within this study [2,50]. However, the best possible technology currently available for hormone determination in a larger study setting was used in the present study. Third, there is no information about the phase of the menstrual cycle the female subjects are in [52], but recruiting subjects in a population study such as LIFE Child ensures that these variables are randomly distributed.

Even if the loss of tooth structure is a major clinical sign of bruxism, it is not itself sufficient to make a definitive diagnosis of bruxism [6]. Tooth wear, as a diagnostic value of bruxism shows lower values of diagnostic specificity than the use of instrumental approaches [8,53]. Additionally, the amount of tooth wear is not related to the severity of the bruxing process because several risk factors may interact with each other [54]. Besides the relation of sex hormones and tooth wear, further studies could investigate if there is a relation between sex hormones and bruxism. The use of reliable bruxism diagnostic procedures could increase the quality of future studies. Lavigne et al. recommend a taskforce to develop a homogeneous assessment of sleep bruxism with new technologies, such as sensors or artificial intelligence, so examinations are better methodologically coordinated [7].

There is currently no consensus on which hormone should be studied in the context of bruxism. Recent studies have contested the long-prevailing belief in a direct association between bruxism and the stress hormone cortisol that is usually isolated from the saliva [27,55]. Because nightly bruxism is especially hard to influence, research additionally evaluates gonadal hormones and their association with sleep bruxism. Effects are described especially for advanced-aged men [56] and women during menopause [57]. It is suggested that the two major female sex hormones, oestrogen [58] and progesterone [59], play an important role concerning the quality of sleep [60]. Since bruxism originates in childhood and in adolescence, an investigation of these relationships with the onset of reproductive age, as it is carried out in the present study, seems more appropriate than after it has ended. The level of testosterone that was examined in the present study is subject to fluctuations throughout the day, and it decreases significantly between 7 and 9 a.m. [34]. This is why most authors recommend early morning testing [61,62,63]. In the present study, fasting blood sampling was performed in the morning. Thus, an extensive standardization of the measured values was achieved.

Many risk factors besides hormones can trigger bruxism. In a case-control study, a consideration of additional risk factors such as caffeine, alcohol, sleep problems, stress, or anxiety [34] would be relevant. However, recruiting subjects in a population study such as the present LIFE Child study ensures that all of these variables are randomly distributed [43,44]. Further, it is unlikely that those factors influence the association between tooth wear and hormones in the sense of a confounder. This would mean that caffeine influences the release of sex hormones. Indication for alcohol as a confounder of steroid hormones do exist, but limitation is given due to a very small study group (only 12 test persons), as carried out by Kumari et al. [64]. It is much more likely to expect an influence in subjects taking hormones (e.g., girls taking oral contraceptives). That is the reason those test persons were excluded from our survey, with age, sex, BMI, and orthodontic treatment essential influencing factors being considered in the survey. The present study observed that male children and adolescents are more often affected by tooth wear than female adolescents. This result corroborates the findings of Sousa et al., 2018 [17], Drumond et al. 2018 [14], Souto-Souza et al., 2020 [15], and Guo et al., 2018 [16]. The difference in sex distribution develops first in adolescence as seen in Figure 2. Data on the frequency and the prevalence described by Sousa [17] go hand in hand with our results. Sousa described a frequency rate of 22.2% in adolescents. In the present study, a percentage of 15.3 among all participants aged between 10 and 18 was described. Within the 10- to 13-year-old children, 9.4% were affected by tooth wear, whereas it applied to 25.5% of youth aged 14 and older.

As participants of higher compliance and social inclusion are more likely to take part in that kind of study [65], a shift toward subjects that show higher socioeconomic status scores could potentially appear and could therefore limit the representativity of the study. However, the effect of the latter is minimal because age, Tanner stage, and hormone levels do not show a correlation with social background. Only the fact that children with higher socioeconomic status scores are more often orthodontically treated might play a role [66]. Similarly, BMI tends to be higher with low social economic status [67,68]. All participants were recruited from Leipzig and the surrounding area, which may restrict the external validity of the tooth wear prevalence data obtained. Apart from socioeconomic status, the demographics of Leipzig (age structure, percentage of Germans with a migration background, number of employees, and employment status) mostly coincide with the entire country. Therefore, it is reasonable to assume that Leipzig is still quite representative of Germany [69].

## 5. Conclusions

In conclusion, the results of the current study revealed a positive association between serum levels of testosterone (index of free androgen) and tooth wear in male adolescents. Therefore, the higher level of sex hormones could be an accompanying factor for this phenomenon. The observed effect was independent of age, sex, and other possible confounding factors such as BMI or orthodontic treatment. This study contributes positively to the understanding of the aetiology of bruxism in the current scientific debate. Therefore, it represents a clinical benefit in an improved dentist–patient communication. The dentist can explain that tooth wear occurs more likely in boys and that it may be accompanied by a higher testosterone level in the affected person.

## Figures and Tables

**Table 1:** Comparison of male and female subjects with or without tooth wear regarding influential variables, statistically significant values are in bold.

	Males Total (*n* = 491)	Tooth Wear (*n* = 86)	No Tooth Wear (*n* = 405)	*p*	Females Total (*n* = 493)	Tooth Wear (*n* = 65)	No Tooth Wear (*n* = 428)	*p*
**Age (mean** **±** **SD) ^b^**	13.0 ± 2.1	14.4 ± 2.1	12.7 ± 2.0	**<0.001**	13.1 ± 2.1	14.1 ± 2.0	12.9 ± 2.1	**<0.001**
**Age group% (N) ^a^**								
**10–13 year**	100 (320)	10.3 (33)	89.7 (287)	**<0.001**	100 (300)	8.3 (25)	91.7 (275)	**<0.001**
**14–18 year**	100 (171)	31.0 (53)	69.0 (118)		100 (193)	20.7 (40)	79.3 (153)	
**BMI** **^2^** **(mean** **±** **SD)** **[kg/m^2^] ^b^, *n* = 978**	21.2 ± 5.5	22.0 ± 5.3	21.0 ± 5.5	n.s. ^1^	21.4 ± 5.9	23.2 ± 6.8	21.2 ± 5.7	**<0.05**
**BMI adj. ≥ 0% (N) ^c^, *n*** **= 978**	58.7 (288)	61.6 (53)	58.0 (235)	n.s.	58.8 (290)	66.2 (43)	57.7 (247)	n.s.
**Tanner stage ^b^, *n*** **= 726**	2.6 ± 1.4	3.5 ± 1.4	2.4 ± 1.3	**<0.001**	3.3 ± 1.3	3.9 ± 1.1	3.2 ± 1.3	**<0.001**
**High SES** **^3^ % (N) ^c^** **, *n*** **= 785**	28.3 (139)	31.4 (27)	27.7 (112)	n.s.	29.4 (145)	20.0 (13)	30.8 (132)	n.s.
**Orthodontic treatment% (N) ^c^, *n*** **= 892**	33.6 (165)	43.0 (37)	32.0 (128)	n.s.	46.2 (228)	49.2 (32)	45.8 (196)	n.s.
**Testosterone (mean** **±** **SD)** **[nmol/mL] ^b^, *n* = 488**	7.3 ± 7.8	12.0 ± 7.6	5.5 ± 7.2	**<0.001**	0.75 ± 0.5	0.88 ± 0.5	0.71 ± 0.5	**<0.05**
**Oestradiol (mean** **±** **SD) [pmol/l] ^b^, *n* = 510**	44.1 ± 32.9	57.4 ± 35.8	39.3 ± 30.5	**<0.001**	236.9 ± 284.0	288.5 ± 293.6	222.9 ± 280.4	n.s.
**SHBG** **^4^ (mean** **±** **SD)** **[nmol/l] ^b^, *n* = 565**	61.2 ± 40.4	44.3 ± 26.5	66.4 ± 42.5	**<0.001**	63.1 ± 36.1	57.8 ± 40.8	64.5 ± 34.8	n.s.
**FT** **^5^ (mean** **±** **SD) ^b^, *n* = 439**	2.3 ± 2.7 × 10^−1^	3.7 ± 2.7 × 10^−1^	1.7 ± 2.6 × 10^−1^	**<0.001**	1.9 ± 2.2 × 10^−2^	2.5 ± 2.1 × 10^−2^	1.7 ± 2.2 × 10^−2^	**<0.05**

^a^ row percent ^b^ *t*-test ^c^ chi- square test ^1^ n.s. = non- significant ^2^ BMI = body mass index ^3^ SES = socioeconomic status ^4^ SHBG = sex hormone- binding globulin ^5^ FT = free testosterone.

**Table 2:** Multivariable analyses of risk for tooth wear in male and female adolescents.

Variables	Males			Females		
	OR ^1^	95% CI ^2^	*p*	OR ^1^	95% CI ^2^	*p*
**Free Testosterone (per unit)**	1.06	1.01–1.12	**<0.05**	1.05	0.52–2.14	n.s.
**Age (per year)**	1.29	1.04–1.59	**<0.05**	1.32	1.08–1.61	**<0.05**
**BMI adjusted (0)**	0.93	0.49–1.79	n.s.	0.74	0.37–1.50	n.s.
**Orthodontic treatment**	1.36	0.70–2.67	n.s.	1.17	0.61–2.28	n.s.

^1^ Odds ratio ^2^ Confidence interval.

## Data Availability

Not applicable.
